# Supplementary material for: Complex‐centric proteome profiling by SEC‐SWATH‐MS
Source: Mol Syst Biol. 2019 Jan 14;15(1):e8438. doi: 10.15252/msb.20188438 (PMC6346213; doi:10.15252/msb.20188438)
Supplement: Supplementary file 8 — Dataset EV7 [file MSB-15-e8438-s008.zip › feature_plots_string/Q13415.pdf]

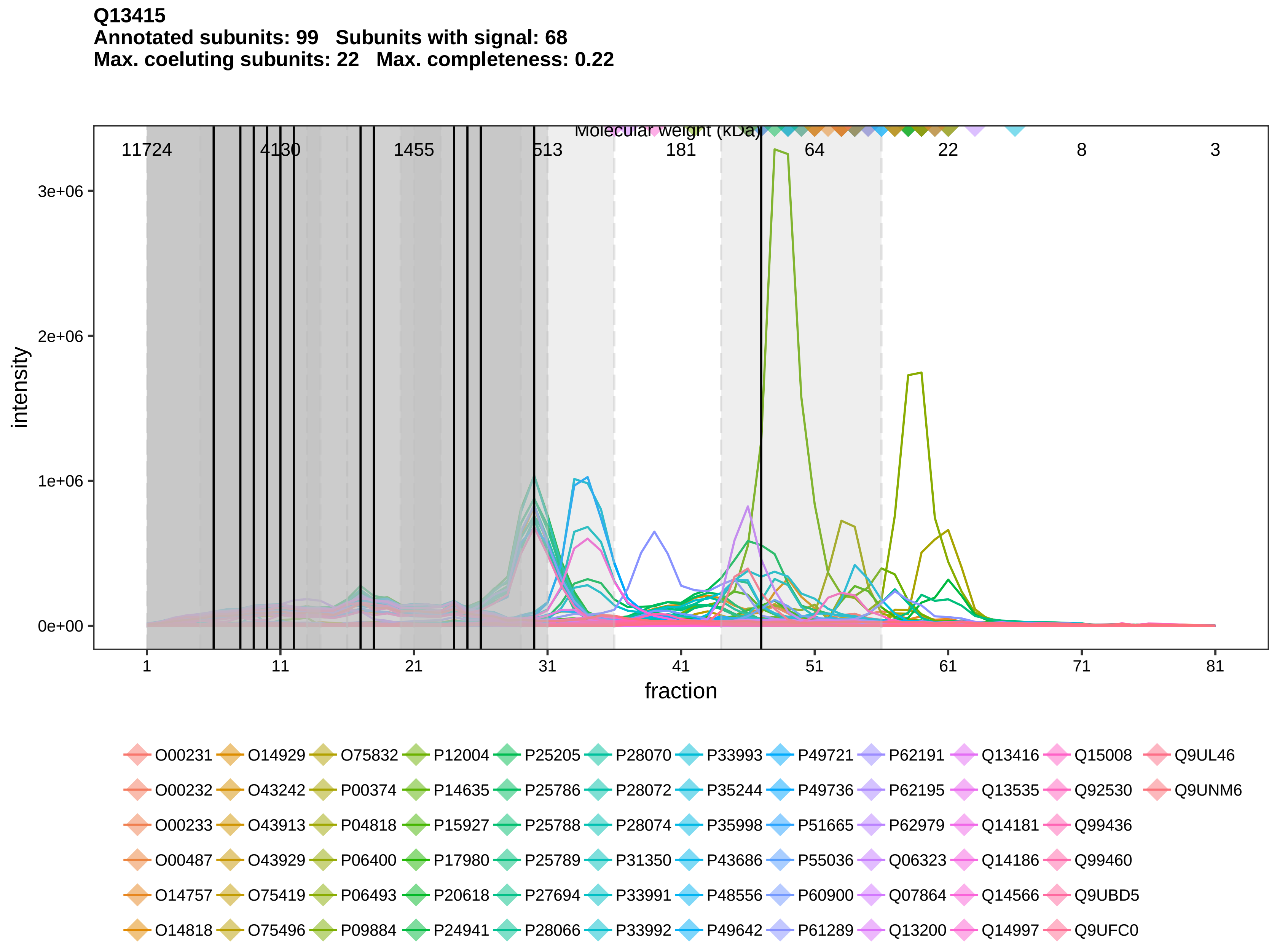
Q13415
Annotated subunits: 99 Subunits with signal: 68
Max. coeluting subunits: 22 Max. completeness: 0.22
Molecular weight (kDa)
11724
4130
1455
513
181
64
22
8
3
intensity
3e+06
2e+06
1e+06
0e+00
fraction
1
11
21
31
41
51
61
71
81
O00231
O00232
O00233
O00487
O14757
O14818
O14929
O43242
O43913
O43929
O75419
O75496
O75832
P00374
P04818
P06400
P06493
P09884
P12004
P14635
P15927
P17980
P20618
P24941
P25205
P25786
P25788
P25789
P27694
P28066
P28070
P28072
P28074
P31350
P33991
P33992
P33993
P35244
P35998
P43686
P48556
P49642
P49721
P49736
P51665
P55036
P60900
P61289
P62191
P62195
P62979
Q06323
Q07864
Q13200
Q13416
Q13535
Q14181
Q14186
Q14566
Q14997
Q15008
Q92530
Q99436
Q99460
Q9UBD5
Q9UFC0
Q9UL46
Q9UNM6
